# Influence of subchondral bone density on intra-articular stresses due to fixation hardware instrumentation and removal: A biomechanical cadaver study

**DOI:** 10.1016/j.injury.2026.113121

**Published:** 2026-02-16

**Authors:** Daniel E. Pereira, Kaitlyn S. Broz, Michelle Gosselin, Joshua D. Namm, Erin L. Hofer, Eric R. Barnard, Donald A. Aboytes, Simon Y. Tang, Anna N. Miller

**Affiliations:** a Department of Orthopaedic Surgery, Washington University in St. Louis, MO, USA; b Department of Orthopaedic Surgery and Musculoskeletal Research Center, Washington University, Saint Louis, MO, USA

**Keywords:** Tibial plateau fracture, Knee osteoarthritis, Knee contact stress, Biomechanical loading study

## Abstract

**Purpose:**

Tibial plateau fractures are often surgically treated to restore native joint congruity and articular alignment. While these injuries portend an increased risk for end stage knee osteoarthritis, it is unknown whether the fixation constructs contribute to the development of osteoarthritis by influencing articular stress distribution following instrumentation.

**Methods:**

We conducted a cadaver study measuring resultant intra-articular stresses of the native knee due to physiological levels of ex-vivo loading, after instrumentation with plate and screw fixation, and after implant removal. To account for variable subchondral bone density, we used 3D printed bone with osteoporotic and normal cancellous bone volume fraction, and SawBones where there is no appreciable cancellous bone.

**Results:**

There was no statistical difference in peak, average, or total contact pressures following implant fixation and removal from the preimplantation articular pressure states in all loads and all models (*p* > 0.05). There was also no difference between the pressure changes of the cadaveric and Sawbones models. There were statistically significant pressure changes between cadaveric and 3D printed models following fixation, however these changes were within previously described physiologic loads (<10 MPa).

**Conclusions:**

Subchondral instrumentation of tibial plateau fractures did not materially alter articular pressures. These findings suggest that the development of end-stage knee osteoarthritis may not be a result of altered biomechancial stresses from the instrumentation. Further, elective removal of implants is not supported by biomechanical reasons alone to reduce future risk. Supplementing cadaveric studies with patient-specific models while tuning variables can enhance the fidelity of these investigations.

**Statement of Clinical Relevance:**

The findings may guide surgeons in their operative indications and clinical decision making as well as guide future biomechanical research on periarticular implant effects.

## Introduction

Tibial plateau fractures are common periarticular injuries that frequently require operative fixation and complex clinical decision making and technical ability in order to achieve long term favorable patient outcomes [1–4]. These fractures are frequently a result of high energy mechanisms such as motor vehicle collisions and are commonly managed surgically to restore native proximal tibia anatomy and joint congruity [1,3]. While operative intervention for tibial plateau fractures often results in excellent clinical outcomes, a significant proportion of patients subsequently develop post-traumatic arthritis which may require total knee arthroplasty (TKA) [3–6]. Recent advances by Resch et al. do demonstrate a possible decrease in postoperative TKA despite the presence of osteoarthritis; however, the trend is worse in obese patients [6]. A recent matched, population-based cohort study found that 7.3 % of patients with operatively fixed tibial plateau fractures went on to develop end-stage osteoarthritis within ten years, five-fold increase compared with the general population. Contemporary research in 2025 confirms this trend as Kaser et al. demonstrate both short and long term sequelae of development of post-traumatic arthritis in this population [4,5]. Although the nature of the injury has a clear correlation with the development of post-traumatic arthritis, it is also important to understand how our surgical interventions may contribute.

Variable contact stress in the tibiofemoral joint has been shown to result in the development of osteoarthritis in settings of malunion, instability, and trauma by mechanical and chemical injury [7–9]. Further, surgical options to reduce or balance contact stress in the knee joint have been shown to slow the progression and symptoms of osteoarthritis, such as in the setting of femoral or tibial osteotomies to unload specific alignments [10,11]. In the ankle joint, it has been suggested that periarticular hardware placement results in elevated articular contact forces, which are hypothesized to result in cartilage degeneration over time [12–16]. In addition, in cadaveric studies, removal of hardware results in a decrease in contact stresses in the ankle joint, with symptomatic improvement in clinical studies [13,17]. For example, Jung et al. demonstrated that routine removal of articular hardware, such as following ankle fixation, may actually result in patient satisfaction; particularly for symptomatic hardware [17]. In tibial plateau fractures, it is understood that anatomic reduction is a critical strategy to mitigate incongruent knee articular contact stresses across the joint. However, it is not known whether the tibial plateau instrumentation itself contributes to articular contact force distortion from baseline. It is also not known whether removal of hardware results in a return to native contact stresses across the knee joint. Removal of hardware is somewhat controversial but has been shown to result in good clinical outcomes in patients with tibial plateau fractures and in ankle fractures [2,17]. We currently do not know definitively whether periarticular hardware fixation in tibial plateau fractures influences the development of end stage knee arthritis following fracture healing and whether staged removal would result in less altered articular stressors.

For these reasons, this study seeks to investigate alteration of joint forces after both placement and removal of plate and screw fixation in proximal tibias using three different biomechanical models. Using a cadaveric, Sawbones, and three-dimensional (3D)-printed models, articular contact pressures were measured following the reduction of osteotomized tibial plateaus and subsequent removal of implants [18–22]. Zdero et al. as others have recently promoted the need for and reliability of these biomechanical studies, using thin articular sensors under dynamic loads to assess surface articular pressure changes over time [22].

## Materials and methods

We first investigated the effects of peri‑articular hardware on knee articular contact pressures in cadaveric tissues. To better understand the influence of subchondral bone volume fracture on the intra-articular pressures, we examined sawbones and 3D-printed tibia constructs where we were able to control the subchondral bone density. These constructs were utilized to augment and compare findings between traditionally used biomechanical specimens and to qualitatively compare the research utility and cost of each. These studies were analyzed with a Tekscan 4015 intra-articular pressure sensor map [20, 21,23–25]. (Appendix 1) Locations of the femoral and tibial implants were marked and were consistently fixed to the loading construct to ensure standardization.

### Sample preparation and loading

#### Cadaver knees

The cadaveric biomechanical study was performed with specimens obtained through a university-associated body donation program. Exclusion criterion included a history of extremities with prior surgeries to the knee or obvious gross deformity to the joint. Additionally, those samples that exhibited Stage 3 or 4 osteoarthritis (cartilage erosion, cortical wear, osteophyte formation) from a macroscopic examination of the articular surface were excluded. Applying these criterion, one lower extremity from each of eight fresh frozen cadavers (2 M / 6 F) were obtained from donors with an average age of 67 (52–79).

**Fig. 1.**
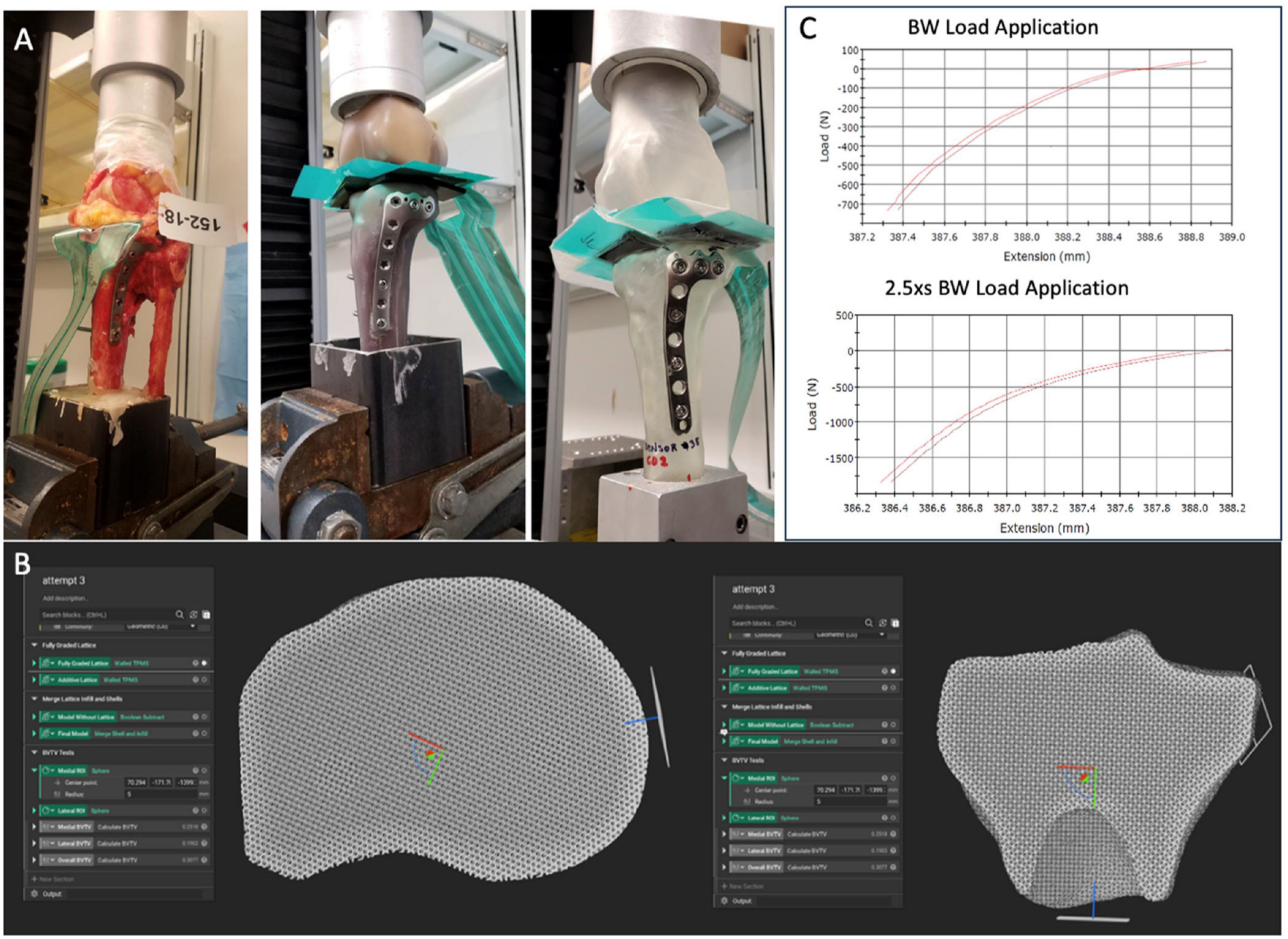
Experimental set-up and load analyses. A. Specimen set-up: Instrumented cadaver, sawbone, and 3D printed tibia with pressure sensors in the joint fixed on the Instron machine. B. Rendering of cancellous bone within 3D printed tibia depicting cancellous bone properties including increased density adjacent to the cortex, a transverse cross-section (Left) and a coronal cross-section (right). C. Example stress-strain curves for BW and 2.5xs BW loading conditions, two tests are shown for each loading condition, a negative value of (N) correlates with a positive application of force at the joint surface. Minimal deformation was noted with load application.

The cadaveric limbs were thawed, potted, and prepared for sensor analysis as described in similar biomechanical models for the tibiofemoral joint (Appendix 1) [20,21,23–25]. Specifically, as Chen et al. suggest, the model was created to be normalized across the board both in initial engineering and in load implementation; for standardization [25]. The Tekscan pressure sensors (Tekscan Inc; Boston, MA) were placed in the medial and lateral compartments and the extremities were taken through several loading sessions to analyze articular stress (Fig. 1). We utilized three loading conditions: load equal to standardized body weight, 2.5 times body weight, and 5 times body weight. A body weight of 75 kg was chosen for the female specimens and 88 kg was chosen for the male specimens, based on current CDC normative data, in order to standardize the load seen by the articular surface.

#### Sawbones and 3D printed models

In order to better understand the influence of subchondral bone, we utilized Sawbone models that do not have appreciable cancellous bone in the tibial plateau (Sawbone SKU 3973, 4th Gen, Composite, 17 PCF Solid Foal Core, Medium). Similarly to the cadaveric sessions, six identical Sawbones (Pacific Research Laboratories, Vashon, WA) femurs and tibias were prepared, mounted with Tekscan sensors, and loaded through several loading sessions to analyze articular stress [24,25]. Chen et al. recently published similar analysis programs using these sensors for the knee articulation under dynamic load, affirming the need for standardization in analysis which was part of the impetus for the utilization of sawbones and 3D printed models [25]. Rubber pads were utilized to simulate cartilage and the material used was selected to be close to articular cartilage thickness (~3 mm) and Young’s Modulus (2–5 MPa). Multiple tests with different materials were completed to arrive at the final rubber material used in the final loading tests (Appendix). Two loading conditions were used in each test: body weight and 2.5 times body weight, using the average body weight of a female as described for the cadaver study.

To create knee models with subchondral bone, CAD files were designed in nTopology to recapitulate the biomechanical integrity and structure of human osteoporotic knees. A CT scan from a healthy adult female was used for baseline bone geometry and cortical bone morphology to standardize the location of instrumentation and hardware fixation (Fig. 1) [26]. Using the bone volume parameters, a bone volume to total volume (BV/TV) ratio was created to simulate healthy and osteoporotic bone. These knee models were then taken through the loading parameters with the Tekscan sensors in place (Appendix 1, Fig. 1). In total, three models were designed for each loading study session.

### Instrumentation and implant removal

#### Cadaver.

A 6-hole lateral proximal tibial locking plate was inserted based on routine implant placement principles and the manufacturer guidelines. Instrumentation location was standardized. The same number of screws were placed in each specimen: all three screws in the proximal row, one oblique screw, and three diaphyseal screws. We utilized a combination of locking and cortical screws with the proximal 4 screws locking and the distal 3 screws cortical. The plate was fixed 5 mm distal to the joint line as measured after instrumentation in each specimen, however cadaver specimen anatomy contributed to some amount of variability in placement. The distance of the peri‑articular hardware was also measured after removal using calipers to measure the distance from the screw holes to the tibial plateau.

After instrumentation, the specimens were placed back onto the load frame and were cycled through testing conditions with the same protocol as the control/pre-instrumentation condition. After the initial load was applied, the pressure sensor was calibrated by research personnel to replicate the pressure map equivalent to the pre-instrumentation condition. After this loading data was obtained and recorded, the plate and screws were removed, and the specimens were once again cycled through the same testing conditions post-instrumentation.

#### Sawbones and 3D printed tibiae.

The Sawbones tibias were fixed in the same plate and screw configuration as the cadaveric tibias. After instrumentation, the specimens were placed back into the Instron machine and were cycled through testing conditions with the exact same protocol as the pre-instrumentation condition. After the preload was applied, the pressure sensor was adjusted to obtain a pressure map equivalent to the pre-instrumentation condition. Contact pressures were recorded for all loading conditions with instrumentation on and following instrumentation removal.

Both the osteoporotic and ‘normal’ 3D printed knee models were instrumented using the same method described above for the cadaver samples. Plate placement was standardized across every tibia as they were identical in bone topography. After instrumentation, the specimens were placed back into the Instron machine and were cycled through testing conditions with the same protocol as the pre-instrumentation condition. After the preload was applied, the pressure sensor was adjusted as needed to obtain a pressure map equivalent to the pre- instrumentation condition. Contact pressures were recorded for all loading conditions with instrumentation on and following instrumentation removal.

#### Analysis.

**Fig. 2.**
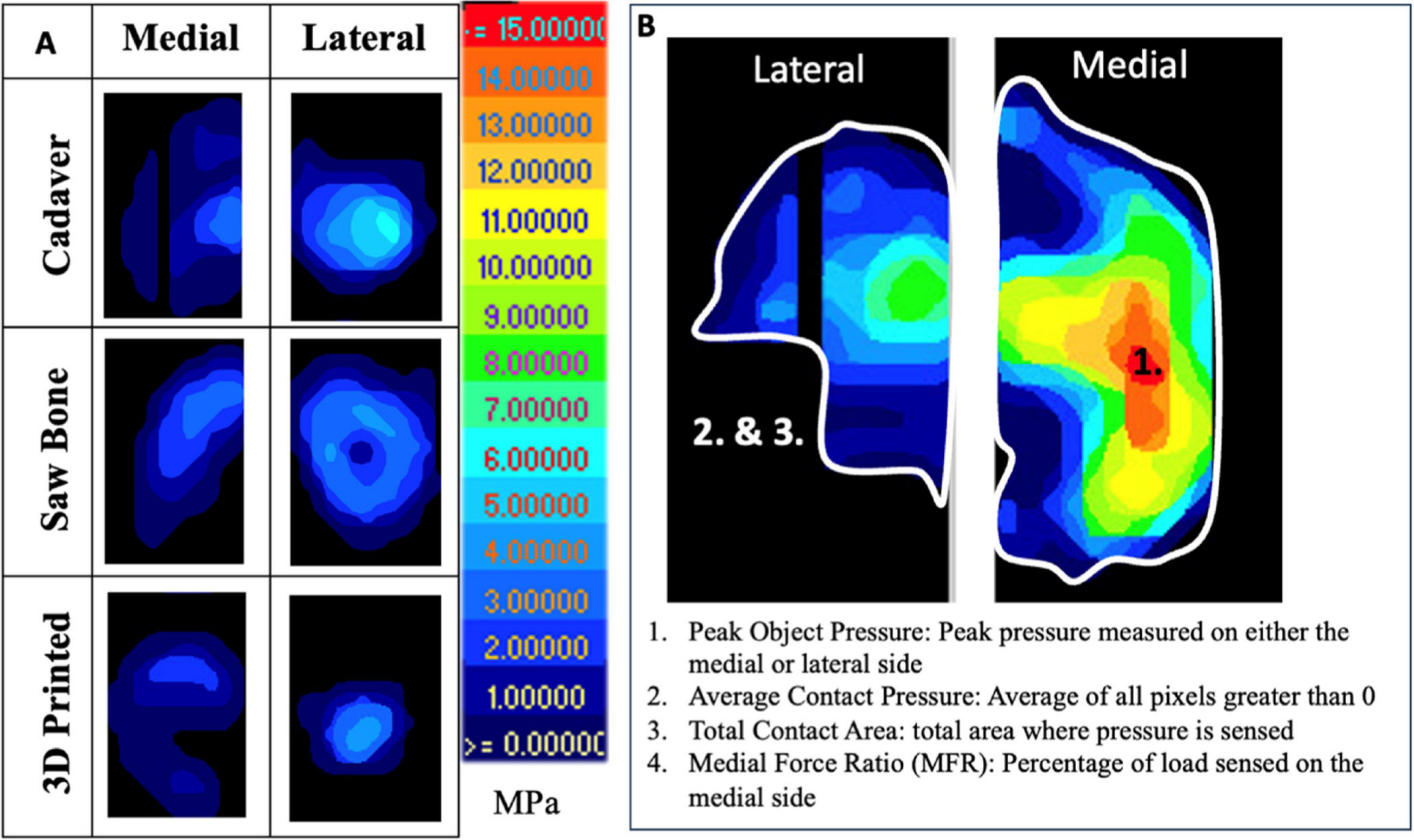
Pressure map analyses. A. Representative pressure maps for the medial and lateral condyles from the cadaver study, sawbone study, and 3D printed tibia study. B. Example Pressure map depicting areas used to calculate the (1) peak object pressure, (2) average contact pressure, (3) total contact area, and (4) medial force ratio.

The data for all the above studies were analyzed in the I-scan software (Tekscan Inc; Boston, MA). Tekscan sensor calibration and degradation analysis was also controlled throughout the testing process (Appendix). Pressure maps were averaged across the entire capture time after load application for only the first load session for each condition (Fig. 2). Subsequently, the peak contact pressure, average contact pressure, medial force ratio (MFR), and total contact area were recorded for each loading condition (Fig. 2).


$$MFR\;=\;\left(\frac{Medial\;Force}{Medial\;Force\;+\;Lateral\;Force}\right)\;*\;100$$

#### Statistical analysis.

A one-way repeated measures ANOVA was performed in Prism 9.5 (GraphPad, San Diego, CA) to test the effects of instrumentation and removal in the peak contact pressure, average contact pressure, MFR, and total contact area. A 3-way repeated measures ANOVA was used to evaluate the effects of loading pressures on contact pressures. In the 3D printed samples a two-way repeated measures ANOVA was also used to test for effects of the bone volume to total volume ratio on these outcomes.

## Results

### Cadaver

**Fig. 3.**
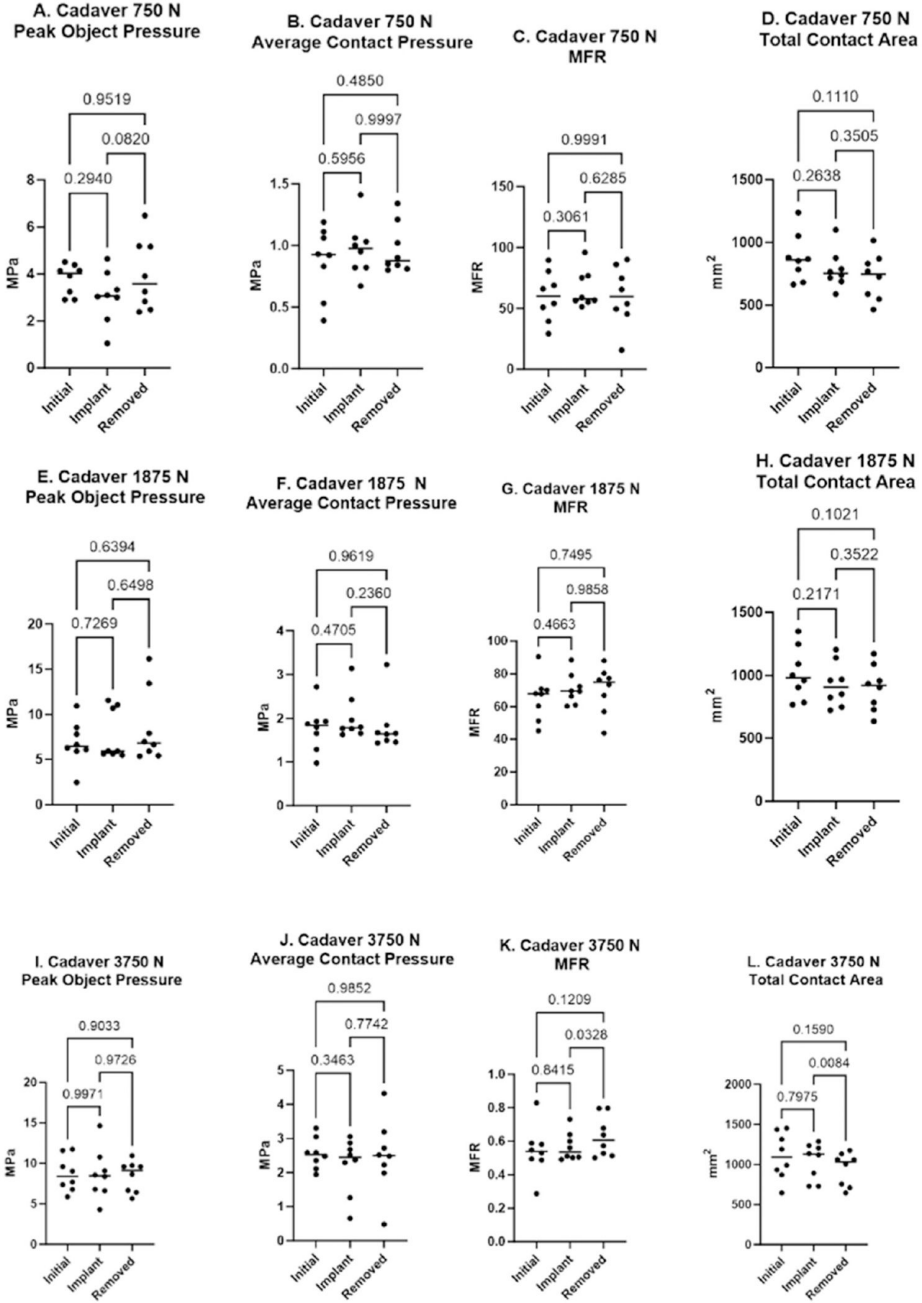
Cadaver pressure measurements. Cadaver pressure measurements for BW and 2.5xs BW loading conditions. No significant differences were noted in (A) 750 N peak object pressure, (B) 750 N average contact pressure, (C) 750 N MFR, (D) 750 N total contact area, (E)1875 N peak object pressure, (F) 1875 N average contact pressure, (G) 1875 N MFR, (H) 1875 N Total Contact Area, (I) 3750 N BW peak object pressure, or (J) 3750 N BW average contact pressure. Posthoc comparison p-values are noted on the graphs, all are greater and 0.05. Small changes were noted at 3750 N, (K) MFR was increased after removal compared to the implanted state and (L) total contact area was decreased after removal compared to implanted.

Analysis of peak object pressure, average contact pressure, medial force ratio (MFR), and total contact area in cadaveric tibial plateaus demonstrated no significant differences with instrumentation placement or removal in all loading phases (Fig. 3).

**Fig. 4.**
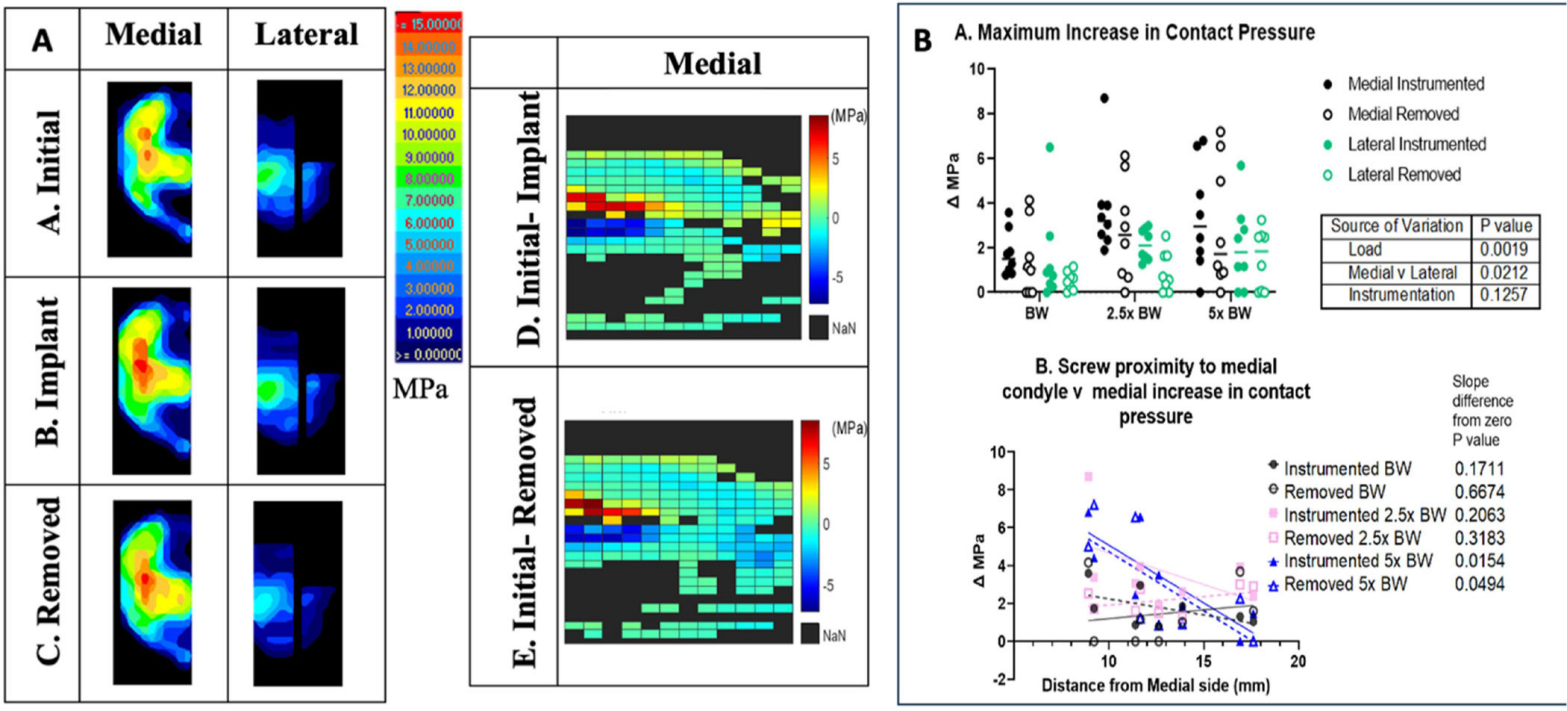
Cadaver pressure maps, difference maps and analysis of increased stress concentration areas. Example pressure maps from the cadaver study where differences were noted from the (A) initial pressure map to the (B) instrumented pressure map and after (C) removal. Difference Maps were made based on these images to detect specific locations of changes in contact pressure, panel (D) depicts positive values indicating areas with increased contact pressure and negative values indicating areas with decreased contact pressure, the same difference maps for the initial minus the removed case is in panel (E). Areas with significantly increased contact pressure were detected with implantation and after removal (A) maximum increase in contact pressure compared to the initial time point is plotted, the magnitude of the increase is different due to loading condition and medial v lateral sides, though no changes due to removal of the instrumentation. (B) At 5x body weight the magnitude of the stress concentrations increased when the screw was placed closer to the medial condyle, no differences were noted at the lower loading conditions.

The focal areas of the tibial plateau revealed that the addition of peri‑articular hardware increased contact pressure in some areas on the tibial plateau compared to the pre-implanted condition (Fig. 4). Hardware removal did not result in recovery of the overall contact pressure or pressure distribution back to pre-instrumentation pressure of these focal areas. The magnitude pressure increase was significantly greater in the medial compartment than the lateral compartment with a greater difference in pressure increase with greater loads (Fig. 4). Additionally, the distance of the screws from the medial side of the tibial plateau the magnitude of the increase in contact pressure was significantly greater when a higher load was applied (3750 N). However, these pressure increases were within the magnitudes (all less than 10 MPa) which have been demonstrated to be normal physiologic levels of pressure on human knees [27–30]

### Sawbones and 3D printed tibiae

**Fig. 5.**
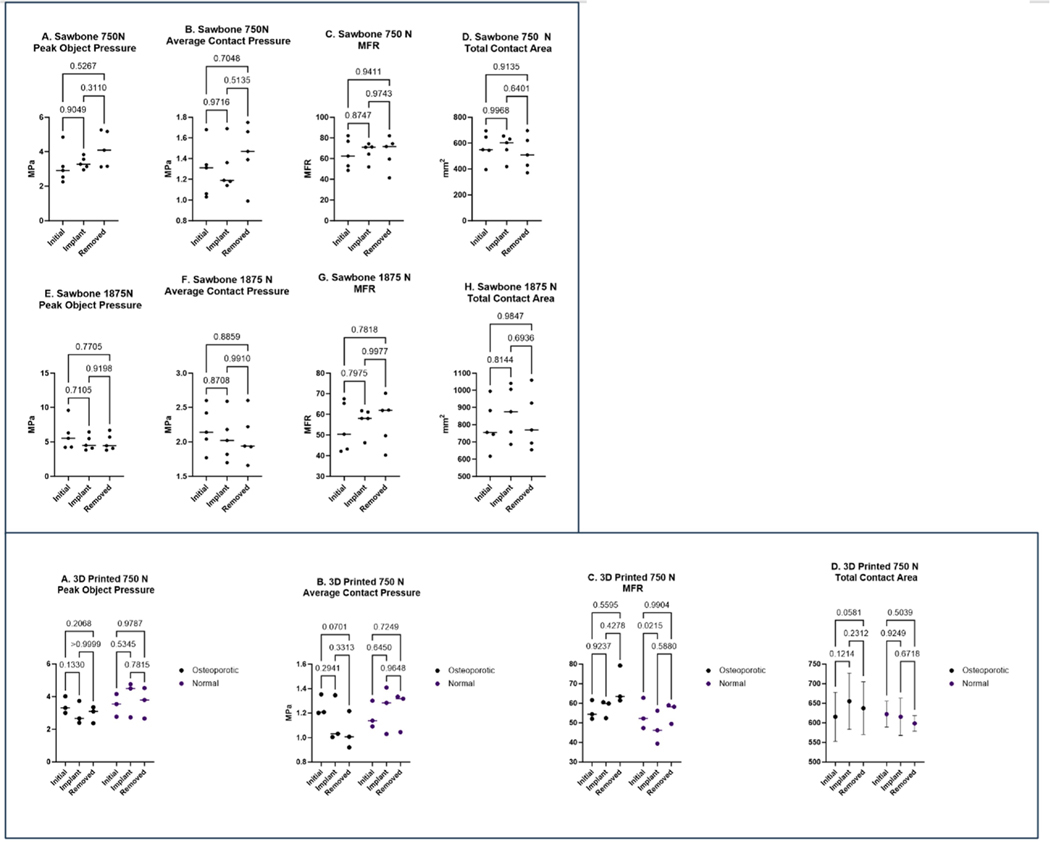
Sawbone and 3D printed Pressure Measurements. A. Sawbone pressure measurements for BW and 2.5xs BW loading conditions. No significant differences were noted in (A) 750 N peak object pressure, (B) 750 N average contact pressure, (C) 750 N MFR, (D) 750 N total contact area, (E)1850 N peak object pressure, (F) 1850 N average contact pressure, (G) 1850 N MFR, or 1850 N total contact area. Posthoc comparison p-values are noted on the graphs, all are greater than 0.05. B. 3D printed pressure measurements for 750 N. No differences noted in (A) peak object pressure, (B) average contact pressure, or (D) total contact area. There is a reduction in the (C) MFR in the “normal” samples that was not noted in the osteoporotic samples.

In the Sawbones series, the peak force, average contact pressure, and peak contact pressure at the tibial plateau were measured in both loading sessions, which demonstrated no statistically significant change across all measures (Fig. 5). This was unique in that there were no statistical differences in any of the outcomes we measured as opposed to our cadaveric and 3D printed models.

There were no significant differences between baseline, instrumented, or post-removal pressures in the normal or osteoporotic groups in peak object pressure, average contact pressure, or total contact area. However, there was a significant difference in the MFR with instrumentation in the “normal” BV/TV samples. This increase in pressure was small (<10 %) and recovered following implant removal (Fig. 5).

## Discussion

This biomechanical study demonstrate that cadaver and Sawbones/3D printed models demonstrated no statistical difference in peak, average, or total contact pressures following implant fixation and removal from the preimplantation articular pressure states in all loads. We did find differences in the Medial Force Ratio (MFR) and focal areas of stress in the 3D printed normal and cadaveric models; these changes were small and generally within normal physiologic measurements [29–31]. For example, Yuh et al. in their recent biomechanical pressure analysis offerend quantitative measures of loading and dynamic activity through the ankle joint and it’s effect on the cellular nature of cartilage cell and structure [15]. This was also found to be significant in the cadaveric models depending on the distance between the most proximal screw and the articular surface in greater loads, however this was still at a level previously shown to be normal physiologically [7–9,27–32]. This is encouraging for Orthopaedic patients, as a second hardware removal surgery to offload the joint after fixation for trauma may not be necessary. Our findings suggest that the implantation and persistence of periarticular hardware in tibial plateau fractures may not contribute meaningfully to articular stress alterations.

Tibiotalar articular pressures significantly increased following transepiphyseal screw fixation, and this improved following removal in one study [10]. This study used a single screw placed at the distal tibia and measured pressure changes across the tibiotalar joint. Our study differs in its utilization of a larger plate and screw fixation construct and in the inherent difference of the knee being a larger joint than the ankle. Additionally, the qualitative component of the pressure changes, specifically how these pressure changes may translate to actual cartilage damage, was not assessed. This is important to note, as Fang et al. and others have noted that even subtle changes in contact pressures and loading environments leads to chondrocyte apoptosis and an increase in deleterious molecular markers [7–9]. While we also found some pressure increases in the cadaveric and 3D printed models, these were not qualitatively interpreted to result in a supra-physiologic stressor across the knee joint. Studies showing improved patient-reported outcomes after hardware removal have not shown a clear connection with articular pressures. Based on current research, the observed increases in focal pressure do not reach the level observed for structural or cellular damage and therefore do not support routine elective removal of hardware for this reason alone [15]. However, these modest increases were not observed over longer periods of time, which may be relevant in patients with indwelling implants for many years. This is clearly an area for future biomechanical and clinical research.

This biomechanical series demonstrates multiple methods of studying joint pressures across the knee. Our findings demonstrated strengths and limitations to each method, which should inform future biomechanical research. Indeed, future investigators seeking to create a biomechanical model for the knee, or the tibial plateau should understand the benefits and drawbacks of each model. The Sawbones series enabled us to investigate the role of the subchondral cancellous bone in load transmission after the addition of screws. We hypothesize that this was due to the inability of the material to simulate normal human bone to the same degree as the other models. The cadaver tissues, while most like living human tibias, are expensive, technically taxing to utilize and highly heterogeneous. In addition, they are more osteoporotic than the average young trauma patient due to the advanced chronologic age of a typical cadaveric tibia. It is also difficult to standardize the level of degeneration, implant placement, and overall size of the bone itself, which has been shown in recent biomechanical research to lead to unreliable measurements [25]. We therefore believe that three dimensional printed tibias demonstrate a promising venue for study of joint pressures, as tibia size, bone quality, and implant placement can be reliably standardized. Further, 3D printed models can also be made to resemble any bone type, including normal or osteoporotic bone, creating greater opportunities for comparison research at a relatively inexpensive price point. In this study, while we find that there are statistically detectable changes in intraarticular pressures following instrumentation in the cadaveric models that do not recover from the removal of the fixation plate, this may depend on the location of the instrumentation, the amount of supporting subchondral cancellous bone, and the cartilage health of the individual. The use of Sawbones and 3D printed models allowed us to remove some of these factors that confound our evaluation of tibial plateau intra-articular stresses.

As with any ex vivo study, these analyses have their limitations. Variabilities in sensor and specimen pressure data and degradation were noted throughout the study but were accounted for in the final analysis (Appendix 1). The distance between the fixation constructs slightly varied between donors due to anatomical variations and could not be directly controlled in the cadaver study. Since the gross morphology of each Sawbones and 3D-printed model were the same, the plates were consistently instrumented at identical sites in each sample. However, the consistency of findings across all three biomechanical models reassures our final conclusions. Torsional forces at the knee as well as varying flexion angles of the knee were not studied in the present investigation due to issues with construct stability, however, these are future areas in need of study. Specifically, recent research has focused on the impact of both BMI, activity level, and molecular markers that can have an effect on the development of arthritis; possibly which can be worsened or exacerbated by these sutble changes [9,27,30]. Finally, while our findings demonstrate mild increase in articular contact stresses (<10 MPa) it does not describe how this increase may compound over time, which has been suggested to lead to chondrocyte apoptosis [7,9]. Indeed, this study should be used as initiative for further biomechanical studies of the effects Orthopaedic fixation may have around joints.

## Conclusions

Removal of instrumentation in tibial plateau fractures did not meaningfully alter articular stress across the tibiofemoral joint in this mixed-method biomechanical study, therefore routine hardware removal cannot be recommended for pure biomechanical reasons. There were significant pressure increases found following instrumentation. These articular pressure changes, however, were at a previously described normal physiologic levels and not at a level expected to result in cartilage damage. After examining cadaveric, Sawbones, and 3D-printed normal and osteoporotic bone to investigate the effect of instrumentation and implant removal on articular pressure, we found that hardware removal would not meaningfully change outcomes in these patients. Three-dimensional printed bone models offered the most reliable and inexpensive biomechanical model to study intra-articular pressure changes in the trauma setting. These findings suggest that removing fixation hardware will not change intra-articular stresses and not likely to alter the risk of future osteoarthritis.

## Supplementary Material

1

Supplementary material associated with this article can be found, in the online version, at doi:10.1016/j.injury.2026.113121.
