# Supplementary material for: Influence of subchondral bone density on intra-articular stresses due to fixation hardware instrumentation and removal: A biomechanical cadaver study
Source: Injury. Author manuscript; Available in PMC 2026 Jul 27. (PMC13404427; doi:10.1016/j.injury.2026.113121)
Supplement: 1 [file NIHMS2167494-supplement-1.docx]

**Appendix 1**

**Cadaveric Preparation**

The cadaveric limbs were thawed to 25 degrees C prior to testing. We transected the lower extremities through the mid shaft of the femur (approximately 15 cm above the joint line) and the mid shaft of the tibia (approximately 20 cm below the joint line) to ensure adequate length for potting and to standardize distance to the joint line. Soft tissue was removed to allow potting and sensor insertion at the tibial plateau. Care was taken to maintain the integrity of the knee static stabilizers (ligaments and menisci). The extremities were potted in poly methyl methacrylate with the knee joint parallel to the ground using a leveling device. Once the potting material cured, 1.5 cm medial and lateral incisions were made through the meniscotibial ligaments to facilitate placement of the intra-articular pressure sensor. We utilized a Tekscan 4015 intra-articular digital pressure sensor (TekScan Inc; Boston, MA) to assess joint line contact pressures (Figure 1). The sensor was placed between two layers of Tegaderm adhesive dressing (3M; Minneapolis, MN) to avoid any fluid penetration into the sensor. Plastic tabs were placed along the edges of the sensor to help facilitate insertion and suture fixation. The pressure sensors were then passed beneath the meniscus, flush with the tibial plateau, without detaching the meniscotibial or meniscofemoral ligaments or their surrounding capsular attachments in the medial and lateral compartments. The plastic tabs were then sutured into the surrounding soft tissue to secure the sensor in place.

Once the intra-articular sensors were secured in place, the extremities were mounted onto an Instron model 5866 servo hydraulic testing machine (Instron Corp; Canton, MA) at full extension (0 degrees). Baseline contact pressure was first measured at each loading condition to serve as control. We utilized three loading conditions: load equal to standardized body weight, 2.5 times body weight, and 5 times body weight.^4^ A body weight of 75 kg was chosen for the female specimens and 88 kg was chosen for the male specimens, based on current CDC normative data, in order to standardize the load seen by the articular surface.^7^ Each testing condition was performed three times to control for any variability. Minimal variability was noted between load applications so only an average of the first load application for each condition is reported.

**Sawbones preparation:**

Six identical biomechanical Sawbones tibias (4^th^ Gen, Composite, 17 PCF Solid Foal Core, Medium) were cut at the midshaft and potted in polymethyl methacrylate with the knee joint parallel to the ground. Femur Sawbones were similarly transected at the mid shaft and potted and fixed on the instron for force application on the tibia Sawbones. To simulate the articular surface, two pieces of rubber with similar mechanical properties to cartilage were attached at the surface of the tibia and femoral condyles. The rubber itself also reduced the sensor noise effects caused by surface friction in the tibial plateau and resulted in pressure readouts like those in the cadaver samples (Fig 1). To simulate the articular surface, two pieces of rubber were placed at the surface of the tibial and femoral condyles, the sheets were cut to the size of the condyles and held in place with compression applied by the Instron so as not to induce any additional materials to the construct, no movement of the rubber was noted throughout the duration of the test. The rubber material was selected to be close to articular cartilage thickness (~3mm) and Young’s Modulus (2-5 MPa). As an additional confirmation a number of rubber materials were tested (3.175mm thick; durometer 50A, 60A, and 70A, equating to 2.5, 3.6, and 5.5 MPa respectively) and the 60A durometer (~3.6 MPa) rubber (McMaster Carr) was selected because it resulted in pressure maps with a contact area and force distribution pattern that most closely resembled the cadaver samples.

Once the intra-articular sensors were secured in place in the medial and lateral knee compartments, the specimens were then mounted into an Instron model 5866 servo hydraulic testing machine (Instron Corp; Canton, MA) at full extension (zero degrees). Baseline contact pressures were obtained using hydraulic testing with an intra-articular pressure sensor. Two loading conditions were used in each test: body weight and 2.5 times body weight, using the average body weight of a female as described for the cadaver study. Six Sawbones tibias were tested with the knee in full extension using the same femur template. The testing conditions did not result in appreciable wear and tear to the femur template between each loading session.

**Three-Dimensional printed models:**

Using a 3-dimensional software analysis and formatting program, 3DSlicer, the cortical bone and medullary cavity of the tibia were segmented from a CT scan of an adult female knee joint. Transposed CT segmentations were exported as meshes and smoothed using an input analysis software, Meshmixer. Meshes were then processed in nTopology where cancellous bone was modeled as the space between the cortical bone and medullary cavity using a gyroid lattice aligned with a vertical framework and utilized a 4mm cell size. Cancellous bone trabecular thickness was characterized by three functions:

1. Cancellous bone that is within 6mm of cortical bone is graded according to proximity to the cortex with a linear multiplier applied to the bone volume fraction in this region that spans 1X-1.5X superficial to deep. For example, if the cancellous bone at a particular site is modeled to have 0.3 BVTV, the wall thickness increases superficially up to 0.6 BVTV where the cancellous bone meets the cortex.
2. Epiphyseal cancellous bone density is graded linearly with the medial and lateral condyles having specified bone densities and the transition between them changing linearly.
3. Lastly, metaphyseal bone volume fraction is graded linearly along the longitudinal axis of the bone with a BVTV multiplier changing from 1X at the metaphysis to 0.8X at the most distal extent of cancellous bone.

All models were printed in the same orientation on the print bed after being exported as .STL files from nTopology. All models were printed using Formlabs clear resin on a Form3 3D printer. Models were washed in a circulating IPA bath for 20 min (in the FormWash). After washing, models were allowed to air dry for 30 minutes. After drying, the models were placed in the FormCure and cured under UV light for 20 minutes at 60*C.

Using the described parameters, cancellous bone volume was controlled to simulate healthy and osteoporotic bone. We then defined the bone volume to total volume ratio (BV/TV) of the subchondral bone to result in osteoporotic (BV/TV = 0.15-0.20) and normal (BV/TV = 0.20-0.30) specimens. To simulate cartilage at the articular surfaces, rubber sheets with thickness and mechanical properties consistent with articular cartilage were placed in the joint. Once the intra-articular sensors were secured in place, the specimens were then mounted into an Instron model 5866 servo hydraulic testing machine (Instron Corp; Canton, MA) at full extension. Following pre-fixation loading and analysis, each of the three specimens were removed from the testing device for instrumentation. 3D printed samples were tested to a load of 750N.

**TekScan Sensor Calibration**

Tekscan sensors were calibrated with a nine-point calibration process from approximately 375N (0.5xs the lowest load “BW”) to approximately 2000N (1.5xs the highest load tested) fit with a regressive curve per TekScan recommendation. Only calibration points with residuals less than 20% were utilized. Sensor sensitivity was set to S-7 (Vtest/Vref@gain: 24/255@1x), equilibration and sensor zeroing was not performed in these tests. Load application was done using the Instron 5866 and calibrations were completed for each test set up and sensor combo. Sensors were used for a maximum of two samples if there were minimal imperfections visible in the sensor after the first use. In some test set ups, a row of sensels would appear with a pressure of zero, inconsistent with the rest of the pressure map, and were subsequently excluded from analysis.

*Controlling for Sensor Degradation*

Importantly, the tekscan pressure sensors degrade over time with greater loading forces. To understand the significance of this change, an additional study was completed on a cadaver to determine the number of loading cycles it would take to alter the pressure and alter outcome measures. These data showed a maximum reduction in peak contact pressure of 0.05 MPa, equal to 20% of the initial contact pressure for that sensel, following 55 loading cycles at 3750N. Therefore, only differences greater than 0.05 MPa or 20% of the initial sensel load were considered for the following analysis and testing did not exceed 25 loading cycles for the Sawbones and 3D printed bones, and 45 loading cycles for the cadaver samples. Additionally, we tested the effects of handing, e.g. removing a cadaver sample from the materials testing system and putting it back. Removal and replacement of the specimen resulted in no changes in peak object pressure, average contact pressure or total contact area, and a slight variance in medial force ratio not exceeding 2%.

*Localized pressure change analysis: Cadaver*

In the cadaver study, we noted visual changes with instrumentation and removal in the pressure maps that were not captured in the averaged data from the whole joint collected from the I-scan software. Consequently, a matlab code was created to register the pressure sensor data from each loading condition. Using this code, the changes in local pressure could be visualized and analyzed from difference maps. This analysis highlighted areas on the tibial plateau where the contact pressure increased and decreased both upon implantation and after removal. These differences exclude any observed difference less than 20% of the initial pressure value or 0.05 MPa whichever was greater to account for sensor degradation. The areas where the maximum pressure increase was observed was determined to be the most clinically relevant and was used for further analysis. Maximum increased areas containing two sensels were averaged, two sensels were used to account for potential noise from a single sensels. The difference maps were used to record the maximum increase in contact pressure (averaged over two sensels) from the pre-implanted to implanted condition and the pre-implanted to post-implantation condition.

Limitations:

First, there is some degradation of the TekScan sensors over time. This limitation was addressed with an additional experiment testing the amount of degradation the sensors see over time, from that experiment we determined a cut off limit of 0.05 MPa, or 20% of the initial selsel value. It is also possible that some variability is introduced when removing the specimen from the Instron machine and replacing it. In the cadaver study, the sensors were sutured to the cadavers and an additional study was done to test that variability. No difference was seen in any contact pressure outcomes, and a slight difference was seen in the medial force ratio. In the Sawbones and 3D printed studies the placement of the specimen on the machines was carefully controlled and marked with tape,; pressure maps were visually confirmed to appear similar after they were replaced on the machine. Furthermore, in the cadaver study, anatomic limitations existed that were more controlled in the Sawbones and 3D printed structures. The screw distance from the tibial plateau varied in the cadaver study and showed alterations in the peak change in contact pressure; in contrast, this was tightly controlled in the Sawbones and 3D printed studies through the use of the locking screws and checking distance of the screw holes to the joint line after removal. Additionally, the time it takes for the cadaver samples to be tested, instrumented, and repeatedly loaded could have resulted in some plastic deformation of the bone that may not be immediately apparent. In addition, there was no radiographic or bone quality data in the cadaver study. The cadaveric series was underpowered to detect differences due to age or sex that we hypothesized would affect the underlying cancellous architecture and the pressure seen at the joint. Consequently, this was the motivation for varying the BV/TV in the 3D printed models.
